# *Citrus aurantium* honey‐mediated gut homeostasis and anti‐inflammation via Thorl/Nprl2‐TORC1 signaling: Network pharmacology and *Drosophila* validation

**DOI:** 10.1002/ame2.70213

**Published:** 2026-06-01

**Authors:** Wenqi Wan, Ruiguang Huang, Shuqiong Cao, Luxia Pan, Wenkai Zhang, Wujun Jiang, Zhiyong Liu

**Affiliations:** ^1^ Science & Technology Center for Experimental Animal of Jiangxi University of Chinese Medicine Nanchang China; ^2^ Clinical Medical College of Jiangxi University of Chinese Medicine Nanchang China; ^3^ College of Animal Science and Technology of Jiangxi Agricultural University Nanchang China; ^4^ Apiculture Research Institute of Jiangxi Province Nanchang China; ^5^ Key Laboratory of Experimental Animal Pathology in Nanchang Nanchang Jiangxi China

**Keywords:** *Citrus aurantium* honey, gastric emptying, intestinal barrier, intestinal inflammation, intestinal motility, network pharmacology

## Abstract

**Background:**

This study aimed to investigate the mechanisms by which *Citrus aurantium* honey modulates gastrointestinal motility, inflammation, and barrier function using network pharmacology and *Drosophila melanogaster* models.

**Methods:**

Ultra‐high‐performance liquid chromatography coupled with Q Exactive high‐field mass spectrometry (UHPLC‐Q Exactive HF‐MS) characterized the chemical profile. Network pharmacology predicted targets and enriched pathways, and molecular docking validated core component–target binding. In lipopolysaccharide (LPS)‐induced *Drosophila* intestinal injury models, functional assays (fecal excretion, intestinal transit, barrier integrity) and mechanistic analyses (reactive oxygen species [ROS] levels, TORC1 pathway–related protein/gene expression) were performed.

**Results:**

Network pharmacology revealed 17 key targets enriched in calcium signaling, cAMP/cGMP‐PKG, and neuroactive ligand–receptor pathways. In *Drosophila*, honey dose‐dependently (0.25% < 0.5% < 1%) enhanced intestinal motility (increased volume of stool and shorter transit time) and reduced inflammation (reduced ROS levels and improved barrier integrity, *p* < 0.01). Mechanistically, honey inhibited TORC1 overactivation by reducing 4E‐BP phosphorylation and regulating Thor/Nprl2 expression.

**Conclusions:**

*C. aurantium* honey exerts gastrointestinal effects via a “multi‐component, multi‐target” mechanism. It modulates smooth muscle contraction through calcium/cAMP/cGMP pathways and alleviates inflammation by suppressing TORC1 signaling, highlighting its potential as a dietary intervention for dysmotility and inflammation.

## Graphical abstract

The study employed a four‐tiered strategy: (1) UHPLC‐FTMS profiling of *Citrus aurantium* honey to characterize its chemical composition; (2) network pharmacology analysis integrating target prediction, protein–protein interaction networks, and KEGG pathway enrichment to identify the Thor1/Nprl2‐TORC1 axis as a key mechanistic pathway; (3) in vitro validation through Drosophila intestinal pH transition experiments and molecular detection of signaling pathway components; and (4) in vivo phenotypic validation including survival analysis, intestinal morphology, barrier function, and inflammatory marker assessment. The findings establish *C. aurantium* honey as a functional food ingredient capable of maintaining gut homeostasis and suppressing intestinal inflammation through TORC1‐dependent mechanisms.

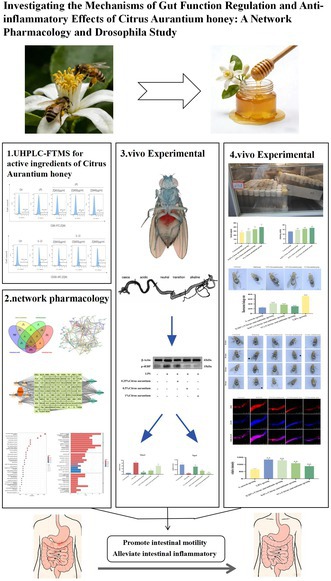

## 1 INTRODUCTION

Gastrointestinal motility disorders (GIMDs) are prevalent clinical conditions characterized by symptoms, including abdominal pain, nausea, vomiting, and diarrhea, which significantly impair patients' quality of life.^1^ Modern medicine attributes GIMD pathogenesis to a complex interplay of psychosocial and physiological factors,^2^ whereas traditional Chinese medicine (TCM) characterizes it via classical syndrome patterns such as “epigastric fullness,” “anorexia,” “gastric reflux,” and “gastric hypotonia.” Notably, dysregulated dietary and negative emotional stimuli are identified as shared pathogenic contributors in both theoretical frameworks.^3^, ^4^

In contemporary societies, the incidence of GIMDs demonstrates an upward trajectory, a trend largely attributable to modern lifestyle determinants such as the accelerated pace of life and a dietary shift toward the prevalent consumption of fast food.^5^, ^6^, ^7^ Epidemiologically, 66% of patients with inflammatory bowel disease (IBD) exhibit comorbid GIMDs. The pathophysiology underlying this high comorbidity is multifactorial, involving synergistic interactions among increased intestinal permeability, immune dysregulation, visceral hypersensitivity (VHS), autonomic dysfunction, and gut microbiota dysbiosis.^8^ Collectively, these mechanisms perpetuate chronic inflammation in IBD and exacerbate intestinal dysfunction severity. Thus, developing targeted therapies to modulate these interconnected pathways is paramount; such interventions are crucial for restoring normal gastrointestinal motility and rebalancing the pathophysiological landscape of intestinal disorders.

Rooted in the core TCM principles of holism and syndrome differentiation, Chinese herbal medicines offer an integrated therapeutic approach to intestinal dysfunction. Their therapeutic potential arises from multicomponent, multitargeted actions, enabling simultaneous regulation of intestinal motility and suppression of pathological inflammation, thereby aligning with the TCM tenet of treating both symptoms and root cause. This therapeutic paradigm is further characterized by high safety and cost‐effectiveness.^9^, ^10^ As documented in classical TCM texts such as the Compendium of Materia Medica, the herb *Citrus aurantium* is recorded to target the “lung, spleen, and large intestine meridians” to “disperse qi stagnation, resolve phlegm, and eliminate food stagnation.” This traditional understanding has been validated by modern pharmacology, which confirms its ability to modulate intestinal function through dual mechanisms. For intestinal dynamics, the modulation of *C. aurantium* constituents (e.g., hesperidin) on smooth muscle contractility is mediated via calcium signaling pathways, underlying the normalization of intestinal transit and amelioration of dysmotility symptoms.^11^, ^12^ At the anti‐inflammatory level, its active components inhibit the activation of inflammatory signaling pathways, including nuclear factor‐κB (NF‐κB); reduce the release of pro‐inflammatory factors such as tumor necrosis factor‐α (TNF‐α) and interleukin‐6 (IL‐6); and alleviate inflammatory damage to the intestinal mucosa.^11^, ^13^ These actions thus establish a mechanistic basis for the application of *C. aurantium* in intestinal dysfunction. *C. aurantium* honey, a monofloral variety derived from the nectar of *C. aurantium* blossoms, exhibits dual therapeutic properties.^9^, ^10^, ^11^ It retains the intestinal‐regulatory properties of the parent herb (*C. aurantium*) while embodying honey's traditional efficacies, including fortifying the spleen, moistening dryness, and harmonizing the stomach.^12^, ^13^ *C. aurantium* honey, traditionally alued in TCM for alleviating digestive symptoms associated with spleen‐stomach deficiency, exhibits mild properties.^11^, ^14^ It retains the intestinal‐modulating effects of the parent herb *C. aurantium*,^8^, ^9^, ^11^ whereas its honey matrix confers protective, demulcent quality that reduces potential irritancy, supporting its suitability for long‐term gut health management. A synergistic interplay between honey‐derived compounds (e.g., flavonoids, phenolic acids)^13^, ^15^ and the active constituents of *C. aurantium* enhances anti‐inflammatory activity. This concerted action provides a mechanistic basis for the honey's efficacy in preventing and treating intestinal dysfunction. Utilizing an integrated approach, this research systematically investigated the role of *C. aurantium* honey in regulating intestinal motility and its underlying anti‐inflammatory mechanisms. The phytochemical profile of the honey was characterized by ultra‐high‐performance liquid chromatography tandem mass spectrometry (UPLC‐MS/MS). Network pharmacology was then employed to predict the interaction pathways between these identified compounds and intestinal function, with key binding affinities being validated through molecular docking simulations. These computational findings were further corroborated by in vivo experiments in a *Drosophila* model. This work thereby establishes a scientific foundation for the potential application of *C. aurantium* honey in preventing and treating GIMDs.

## 2 MATERIALS AND METHODS

### 2.1 Experimental animals

The wild‐type *Drosophila melanogaster* (W1118 stock), obtained from the Experimental Animal Science and Technology Center of Jiangxi University of Chinese Medicine, was housed in an environmental chamber set to 22°C, 55%–60% relative humidity, and a 12/12 h light/dark cycle.

### 2.2 Reagents

*C. aurantium* honey, authenticated and provided by the Jiangxi Apiculture Research Institute (Zhangshu City, Jiangxi Province). Reagents from Beijing TransGen Biotech Co., Ltd. were used as follows: TransZol Up (no.: T2250407); RNA extraction reagent (no.: T1250407); wash buffer 9 (no.: S4250114); clean buffer 9 (no.: S4250112); RNase‐free water (no.: T2250407); 5 × All‐in‐One Reaction Mix for qPCR (no.: R20516); TransScript Uni All‐in‐One Enzyme Mix (no.: Q20824). Reagents from Beijing Solarbio Science & Technology Co., Ltd. were used as follows: lipopolysaccharide (LPS) (no.: L8880); superoxide dismutase (SOD) activity assay kit (no.: 2411012); BCA protein concentration assay kit (no.: 2500080015); 100 × protease inhibitor cocktail (EDTA‐free) in DMSO stock solution (no.: 23JB0817w); 10 × electroblotting buffer (no.: 2312011); non‐fat milk powder (no.: 3550114003). Reagents from Beijing J&K Scientific Co., Ltd. were used as follows: food‐grade Brilliant Blue (BB). Reagents from Biosharp were used as follows: 4% paraformaldehyde universal tissue fixative (no.: 24312112). Reagents from Wuhan Servicebio Technology Co., Ltd. were used as follows: phosphate‐buffered saline (PBS) buffer (no.: GA24100111154); 10 × ice‐bath free fast transfer buffer (no.: GC24110116); *Drosophila* gapdh primers (sense/antisense) (GenBank accession no.: NM_001273184.2); *Drosophila* Thor primers (sense/antisense) (GenBank accession no.: NM_057947.4); *Drosophila* Npel2 primers (sense/antisense) (GenBank accession no.: NM_132946.3). Reagents from GLPBIO were used as follows: anti‐fluorescence quenching mounting medium, 25 mL (containing Triton X‐100); immunofluorescence secondary antibody dilution buffer, 100 mL (containing dihydroethidium [hydroethidine], 10 mg); immunostaining blocking buffer (with DAPI solution, ready‐to‐use, 10 μg/mL); immunostaining primary antibody dilution buffer, 100 mL (containing Phalloidin, 1 mg); PBS buffer (no.: PS0295‐500ML). Reagents from Suzhou Uelandy Biotechnology Co., Ltd. were used as follows: ultra‐sensitive chemiluminescence detection reagent (no.: 250427E01‐65). Reagents from Shanghai Yamei Biomedical Technology Co., Ltd. were used as follows: Tris‐glycine‐SDS electrophoresis buffer (10 ×) (no.: 038C1400); PAGE gel preparation solution (no.: 038B11200); modified coagulant accelerator (no.: 038852000). Reagents from Wuhan Xinsaimai Biotechnology Co., Ltd. were used as follows: ultra‐sensitive chemiluminescence detection reagent (no.: 250427E01‐65); Western blot membrane regeneration solution (no.: 20250318). Reagents from Hangzhou Huidan Biotechnology Co., Ltd. were used as follows: Phospho‐4EBP antibody (no.: A25181). Reagents from Wuhan SanYing Biotechnology Co., Ltd. were used as follows: β‐actin antibody (no.: 81115‐1‐RR); HRP‐conjugated rabbit secondary antibody (no.: RGAR001); TE buffer (no.: 2500055001).

### 2.3 Instrumentation

Electronic analytical balance (model: EX125ZH, Ohaus); electronic balance (model: AUW120, Shimadzu Philippines); microplate reader (model: Varioskan Flash, Thermo Fisher); CO_2_ incubator (model: CB170, Binder); *Drosophila* CO_2_ anesthesia device, Nanchang Jiangzhu; high‐speed refrigerated benchtop centrifuge (model: Allegra 64R, Beckman Coulter); benchtop centrifuge (model: TDZ5‐WS, Luxiangyi); low‐speed centrifuge, Beckman Coulter Suzhou; UHPLC‐Q Exactive HF‐X Fourier transform mass spectrometer, Thermo Fisher; electronic balance (model: New Classic MF MS105DU, Mettler Toledo); general‐purpose electrophoresis system (model: WIX‐EP600, Beijing Weikes); digital deep‐view imaging system, Hangzhou Xiyao; real‐time PCR system (model: LightCycler 96 System, Jiangxi Tongxin); ultramicro fluorescent UV–visible spectrophotometer, Thermo Fisher; electronic blot imaging system (model: Blot, Shanghai Yibote); laser confocal microscope (Leica TCS SP8; Leica7csp8).

### 2.4 Experimental methods

#### 2.4.1 Characterization of bioactive constituents in *C. aurantium* honey using UHPLC‐Q Exactive HF‐MS analysis

##### Sample preparation

A 100 μL aliquot of the liquid specimen was pipetted into a 1.5–mL centrifuge tube, and 400 μL extraction solvent (acetonitrile: methanol = 1:1, v/v) containing 0.02 mg/mL L‐2‐chlorophenylalanine (internal standard) was added. The mixture was vortexed for 30 s, then ultrasonicated at 5°C and 40 kHz for 30 min. After incubation at −20°C for 30 min (for precipitation), the sample was centrifuged at 13 000 × g for 15 min at 4°C. The supernatant was concentrated to dryness under nitrogen stream, resuspended in 100 μL reconstitution solvent (acetonitrile: water, 1:1, v/v), ultrasonication at 5°C and 40 kHz for 5 min, and centrifuged again at 13 000 × g for 10 min at 4°C. The clarified supernatant was transferred to an insert‐equipped injection vial for UHPLC‐Q Exactive HF‐MS analysis.

##### Quality control sample

A quality control (QC) sample was prepared by mixing equal‐volume aliquots of all test specimens. It was analyzed intermittently (after every 5–15 experimental samples) to monitor system stability and evaluate methodological repeatability.

##### Chromatographic conditions

Analyses were performed on a Thermo Fisher UHPLC system with an HSS T3 column (100 mm × 2.1 mm i.d., 1.8 μm) maintained at 40°C. The injection volume was 3 μL. Mobile phase system: (A) water +5% acetonitrile +0.1% formic acid; (B) 47.5% acetonitrile +47.5% isopropanol +5% water +0.1% formic acid. Flow rate was 0.40 mL/min.

##### Mass spectrometry conditions

Mass spectrometric detection was conducted on a Q Exactive HF‐X hybrid quadrupole‐Orbitrap mass spectrometer in data‐dependent acquisition (DDA) mode, with alternating positive and negative ionization. Key parameters: *m/z* range 70–1050, sheath gas flow rate of 50 psi; auxiliary gas flow rate of 13 psi; auxiliary gas heater temperature maintained at 425°C; capillary temperature 325°C; electrospray voltages +3.5 kV (positive mode) and −3.5 kV (negative mode); stepped normalized collision energies 20, 40, and 60 eV; mass resolution 60 000 (full‐scan MS) and 15 000 (MS/MS fragmentation).

#### 2.4.2 Network pharmacology for putative target identification of *C. aurantium* honey

A systematic bioinformatic workflow was implemented to identify the putative targets of *C. aurantium* honey. Canonical SMILES strings and two‐dimensional (2D) structures of the active components were retrieved from PubChem. With *Homo sapiens* designated as the target species, these structural data were input to SwissTargetPrediction to extract all predicted targets with a probability > 0. After data integration and duplicate removal, all predicted targets were annotated and validated using UniProt with standardized gene nomenclature and official identifiers.

#### 2.4.3 Screening of intestinal motility‐related targets via database queries

Targets associated with intestinal evacuation and peristalsis were identified through systematic queries of the GeneCards (https://www.genecards.org/) and OMIM (https://www.OMIM.org/) databases using the search terms “intestinal transit,” “intestinal motility,” and “intestinal peristalsis.” All putative targets meeting the threshold of “score” > 5 were retained. After the removal of duplicate entries, a final set of unique targets related to intestinal emptying and peristalsis was established.

#### 2.4.4 Protein–protein interaction network

To elucidate the common targets through which *C. aurantium* honey modulates gastrointestinal motility, we conducted a comprehensive bioinformatic analysis. We constructed the protein–protein interaction (PPI) network for the shared targets on STRING database (https://string‐db.org/) with a confidence level of 0.4. The PPI graph was subsequently refined using Cytoscape software (version 3.10.3). Core genes were identified as the top‐ranked genes based on degree values.

#### 2.4.5 GO and KEGG pathways enrichment analyses

To elucidate the putative biological functions and signaling pathways through which *C. aurantium* honey regulates intestinal emptying and motility, we performed comprehensive Gene Ontology (GO) and Kyoto Encyclopedia of Genes and Genomes (KEGG) pathway enrichment analyses. The gene symbols of intersecting targets were converted to Entrez IDs using R version 4.5.1 with the following loaded packages: clusterProfiler, org.Hs.eg.db, enrichplot, ggplot2, and pathview. For GO functional analysis, which comprises biological processes (BP), cellular components (CC), and molecular functions (MF), we applied stringent filtering criteria retaining only terms with *p* < 0.05 and false discovery rate–corrected *q* < 0.05. The top 10 significantly enriched terms from each GO category and the top 30 pathways from KEGG enrichment analysis were subsequently selected for visualization.

#### 2.4.6 Network construction of the “bioactive compounds of *C. aurantium* honey–core targets–intestinal motility” axis

An integrated network encompassing bioactive constituents from *C. aurantium* honey, shared protein targets, and intestinal motility‐related disorders was constructed and visualized using Cytoscape software (version 3.10.3). In this network, bioactive compounds, molecular targets, and pathological disorders were represented as individual nodes, with their inherent biochemical and pharmacological interactions designated as edges, thereby illustrating the putative mechanism of action of *C. aurantium* honey.

### 2.5 Molecular docking

Molecular docking was performed to investigate the interactions between the core targets of *C. aurantium* honey and proteins related to intestinal emptying and motility. Initially, we retrieved the three‐dimensional (3D) structure of the protein from the Protein Data Bank (PDB) database (https://www.rcsb.org/) and eliminated water molecules and endogenous ligands from the protein structure. AutoDock Tools (version 1.5.7) was employed for protein hydrogenation and charge calculation. Subsequently, the 2D structures of the bioactive constituents (small molecular ligands) were acquired from the PubChem database (https://pubchem.ncbi.nlm.nih.gov/). AutoDock Tools was used to check the charge balance and rotatable bond of the small molecule. Lastly, Autodock Vina (version 1.1.2) was utilized for conducting molecular docking simulations, and PyMol software (version 3.1) was utilized for image visualization and embellishment.

### 2.6 *D. melanogaster* experiments

#### 2.6.1 Culture media preparation methodology

The basal culture medium was prepared with the following components: corn flour (26.25 g), sucrose (18.75 g), agar (1.875 g), yeast powder (10 g), propionic acid (1.25 mL), methylparaben (3 mL), and distilled water (250 mL). All dry components were first mixed thoroughly in a sterile glass beaker, followed by the sequential addition of liquid components (propionic acid, methylparaben) and distilled water. The mixture was stirred continuously until homogeneous and then dispensed into sterile *Drosophila* culture vials (20 mL per vial) before solidification.

The *C. aurantium* honey‐supplemented medium was prepared starting from the autoclaved basal medium, as described above. To preserve the heat‐labile bioactive components of the honey, supplementation was performed aseptically only after the autoclaved medium had cooled to 40–60°C. Precisely weighed honey was incorporated to achieve final concentrations of 0.25%, 0.5%, and 1% (w/v), followed by thorough homogenization. The resulting honey‐supplemented medium was dispensed into sterile culture vessels, solidified at ambient temperature, and stored at 4°C in the dark until further use.

#### 2.6.2 Preparation of LPS‐containing and *C. aurantium* honey–LPS co‐supplemented media

The LPS‐containing medium was prepared by dissolving LPS directly in the basal medium to a final concentration of 100 μg/mL. After thorough homogenization, the medium was sterilized by autoclaving, dispensed into culture vessels, and allowed to solidify. All aliquots were stored at 4°C in the dark until further use.

For the *C. aurantium* honey–LPS co‐supplemented medium, autoclaved basal medium was first cooled to 40–60°C. Precisely weighed *C. aurantium* honey was then aseptically added to achieve final concentrations of 0.25%, 0.5%, and 1% (w/v), followed by the concurrent addition of LPS to 100 μg/mL. After complete mixing, the medium was immediately dispensed into sterile containers, solidified at room temperature, and stored in light‐proof containers at 4°C.

#### 2.6.3 Experimental *D. melanogaster* grouping and treatment

After randomization, *D. melanogaster* were allocated into four experimental groups (*n* = 18 per group, with a sex ratio of 2:1 [12 females and 6 males]): (1) a blank control group maintained on basal medium; (2) an LPS‐induced inflammatory model group reared on medium containing 100 μg/mL LPS; (3) a *C. aurantium* honey treatment group exposed to medium supplemented with 0.25%, 0.5%, or 1% (w/v) *C. aurantium honey*; and (4) a *C. aurantium* honey preventive intervention group reared on the corresponding honey‐supplemented media coadministered with 100 μg/mL LPS. The sex ratio and group size were designed to approximate natural reproductive clustering while minimizing density‐dependent stress and sex‐specific variability. All groups were maintained under identical controlled environmental conditions, and both phenotypic and molecular indicators were systematically monitored throughout the study.

#### 2.6.4 Observation and detection indicators

##### Fecal pellet counting and quantitative analysis of defecation frequency

After a 3‐day feeding period on 0.5% BB‐supplemented medium, *Drosophila* were transferred to empty vials (*n* = 10 flies per vial) and maintained at 25°C. After exactly 1 h, the number of distinct blue fecal spots on the vial walls was quantified under standardized conditions. The mean fecal pellet count per fly was then calculated for each experimental group.^16^

##### Quantification of *Drosophila* food consumption

Food intake in *D. melanogaster* was quantified using an established protocol. Control flies were maintained on their respective media for 20 days, whereas treatment groups were initially cultured on basal medium for 10 days before transfer to medicated medium for an additional 10 days. On day 21, all flies were transferred to empty vials and subjected to a 1‐h fasting period, followed by feeding with 0.5% BB in 6% glucose solution via capillary feeding. After 5 h, flies were anesthetized with CO_2_ and homogenized in grinding vessels with 200 μL ultrapure water using a mechanical homogenizer. The homogenate was then diluted with 800 μL ultrapure water, centrifuged, and the supernatant was collected. After 30 s of vortex mixing, 200‐μL aliquots were transferred to a 96‐well plate for absorbance measurement at 625 nm. Each experimental group included 20 flies with three independent replicates.^17^

##### Climbing assay

Ten newly enclosed (3‐day‐old) male flies were placed at the bottom of an empty vertical vial. The vial was gently tapped to ensure that all flies were at the bottom, and the timer was started simultaneously. After a precise 11‐s interval, a digital image was taken. The assay was performed with three independent biological replicates. Climbing performance was quantified using the following formula: climbing index (%) = (number of flies at a height ≥ 8 cm/total flies) × 100%.

##### In vivo fluorescent tracer clearance assay

Thirty newly enclosed (3‐day‐old) male flies were first collected and then maintained on 2.4% (w/v) BB FCF‐supplemented medium for 3 days to allow systemic uptake of the fluorescent tracer for subsequent assessment of intestinal integrity. Flies were then transferred to a standard diet, and imaging was performed at selected time points: 0, 1, 2, 3, and 5 h posttransfer. Individuals were anesthetized and positioned in clear imaging chambers, and abdominal fluorescence was documented using a stereomicroscope equipped with a fluorescence module. For each condition, three independent biological replicates were performed. The temporal decay of fluorescence intensity, which correlates with gut motility, was quantified using image analysis.

##### Blue smurf assay

Thirty newly enclosed (3‐day‐old) male flies per replicate were maintained under the conditions previously described for 72 h and subsequently subjected to a 1–h starvation period on moistened filter paper to clear the gut. Flies were then transferred to vials containing filter paper impregnated with a solution of 2.4% BB FCF and 5% sucrose. After a 12‐h feeding period, flies were dissected in ice‐cold PBS to isolate the midguts. The midguts were fixed in 4% paraformaldehyde for 20 min at room temperature, rinsed thrice with PBS, and then mounted in 70% glycerol for microscopic examination.^18^ The blue smurf phenotype, characterized by systemic dye leakage into the hemocoel, was scored visually. The leakage incidence was quantified as the percentage of flies exhibiting the phenotype within the total number of flies assayed per experimental group.

##### Measurement of intestinal ROS levels

Intestines were dissected from adult female *Drosophila* in prechilled PBS using a stereomicroscope. The dissected tissues were immediately incubated with dihydroethidium (DHE) diluted in PBS for 30 min in the dark. After three 5‐min washes with PBS, the tissues were fixed in 4% paraformaldehyde for 30 min in the dark and then washed again as before. For nuclear counterstaining, tissues were incubated with DAPI for 5 min in the dark, followed by three 5‐min PBS washes. Finally, the intestines were mounted in 70% glycerol and visualized using a fluorescence microscope. The mean fluorescence intensity of DHE, which correlates with intracellular superoxide anion production, was quantified from acquired images using ImageJ software (version 1.8.0).

##### Assessment of TORC1 signaling activity using Western blot

Midgut tissues were microdissected from adult *Drosophila* across experimental groups. Total protein was extracted using RIPA buffer, and concentrations were determined using a BCA assay. Equal amounts of protein lysates were resolved using sodium dodecyl sulfate‐polyacrylamide gel electrophoresis (SDS‐PAGE) and electrophoretically transferred onto a polyvinylidene fluoride (PVDF) membrane. After blockade with 5% nonfat milk, the membrane was incubated overnight at 4°C with a primary antibody targeting phospho‐4E‐BP1 (Thr37/46). After washing, the membrane was probed with an HRP‐conjugated secondary antibody for 1 h at room temperature. Immunoreactive bands were visualized using an enhanced chemiluminescence substrate, and band intensities were quantified using ImageJ software. The relative phosphorylation level of 4E‐BP1 was normalized to a loading control and used as a read‐out of TORC1 kinase activity.

##### Quantification of Thor and Nprl2 transcript levels in *D. melanogaster* total RNA extraction

Total RNA was isolated from 7‐day‐old adult *Drosophila* using TRIzol reagent according to the manufacturer protocol.^19^ For each experimental group, flies were anesthetized by brief exposure to CO_2_ and euthanized in 30% hydrogen peroxide solution. Whole flies were homogenized in 1 mL TRIzol on ice using a motorized pestle, followed by incubation at 4°C for 5 min to ensure complete lysis. Chloroform (200 μL) was added, and the mixture was vortexed for 15 s and centrifuged at 12 000 × g for 10 min at 4°C. The upper aqueous phase was transferred to a new tube, mixed with an equal volume of isopropanol, and incubated at room temperature for 10 min to precipitate RNA. The RNA pellet was washed twice with 75% DEPC‐treated ethanol, air‐dried, and eluted in 25 μL DEPC‐treated water. RNA concentration and purity were assessed using a NanoDrop spectrophotometer (Thermo Fisher) by measuring the A_260_/A_280_ ratio.

##### cDNA synthesis

First‐strand complementary DNA (cDNA) was synthesized from 1 μg total RNA using the PrimeScript RT reagent kit (Takara, catalog no. RR037A) in a 20 μL reaction system: 1 μL RNA, 1 μL random hexamer primers (100 pmol), 4 μL dNTP mix (2.5 mmol/L each), 4 μL 5 × RT buffer, 0.5 μL RNase inhibitor (20 U), 1 μL reverse transcriptase (200 U), and RNase‐free water to volume. The reaction was performed at 80°C for 5 min (denaturation), followed by 50°C for 30 min (cDNA synthesis) and 85°C for 5 min (enzyme inactivation). cDNA products were stored at −20°C.

##### Quantitative real‐time PCR

Quantitative real‐time polymerase chain reaction (qRT‐PCR) was performed using SYBR Green Premix Ex Taq (Takara, catalog no. RR420A) on a LightCycler 96 System (Roche). The 20 μL reaction system contained the following: 10 μL 2 × SYBR Green Mix, 0.4 μL forward primer (10 μmol/L), 0.4 μL reverse primer (10 μmol/L), 2 μL cDNA template, and 7.2 μL ddH_2_O. All reactions were run in triplicate. The amplification program was initial denaturation at 95°C for 30 s, 40 cycles of 95°C for 5 s and 60°C for 30 s, followed by a melting curve analysis (60–95°C) to verify amplicon specificity. Primer sequences for Thor, Nprl2, and reference genes (β‐actin, rp49) are listed in Table 1. Relative gene expression was calculated using the 2^(−ΔΔCt) method, with β‐actin and rp49 as dual internal controls.

**TABLE 1:** Primer sequences for quantitative polymerase chain reaction (PCR) analysis of *Drosophila* genes.

Gene	Sequence
*Drosophila* β‐Actin	Forward:5′TTAGCGATGCCAAATGCCAG3′
	Reverse:5′CAGACGAGTCCAATTAGAAGCCA3′
*Drosophila* RP49	Forward:5′AGATCGTGAAGAAGCGCACCAAG3′
	Reverse:5′CACCAGGAACTTCITGAATCCGG3′
*Drosophila* Thor	Forward:5′TGCCCATGATCACCAGGAAG3′
	Reverse:5′CCTCCAGGAGTGGTGGAGTA3′
*Drosophila* Nprl2	Forward:5′TCAAGGATCACATGGTGCCC3′
	Reverse:5′AACGACCCCGTAGTAGACCA3′

All nucleotide sequences used in this study were retrieved from the NCBI GenBank database and commercially synthesized by Wuhan Servicebio Technology Co., Ltd. The specific sequences are provided in Table 1.

Composition of the real‐time PCR reaction mixture is listed in Table 2.

**TABLE 2:** Quantitative polymerase chain reaction (qPCR) formulation for gene expression analysis in *Drosophila*.

Reagent	Volume (μL)
Fast SYBR Green Master Mix	10
cDNA	2
ddH_2_O	6
Forward primer	1
Reverse primer	1
Total reaction volume	20

Realtime‐PCR reaction protocol is detailed in Table 3.

**TABLE 3:** Real‐time polymerase chain reaction (PCR) protocol.

Program step	Temperature (°C)	Time	
Predenaturation	95	10 min	
Denaturation	95	15 s	40 cycles
Annealing	60	1 min	
Extension	72	20 s	
Melting	95	15 s	Generate a melting curve with continuous fluorescence monitoring
	60	1 min	
	95	15 s	

### 2.7 Statistical analysis

Statistical analysis was performed using IBM SPSS Statistics, version 26.0. All continuous variables are presented as mean ± standard deviation. Group comparisons were analyzed by one‐way analysis of variance (ANOVA). Upon observing a significant effect by ANOVA, post‐hoc comparisons between specific groups were conducted using the least significant difference (LSD) test. All figures were constructed using GraphPad Prism, version 9.3.1. Statistical significance was defined as a two‐tailed *p* < 0.05.

## 3 RESULTS

### 3.1 Major components in *C. aurantium* honey identified using UHPLC‐Q Exactive HF‐MS

**FIGURE 1:**
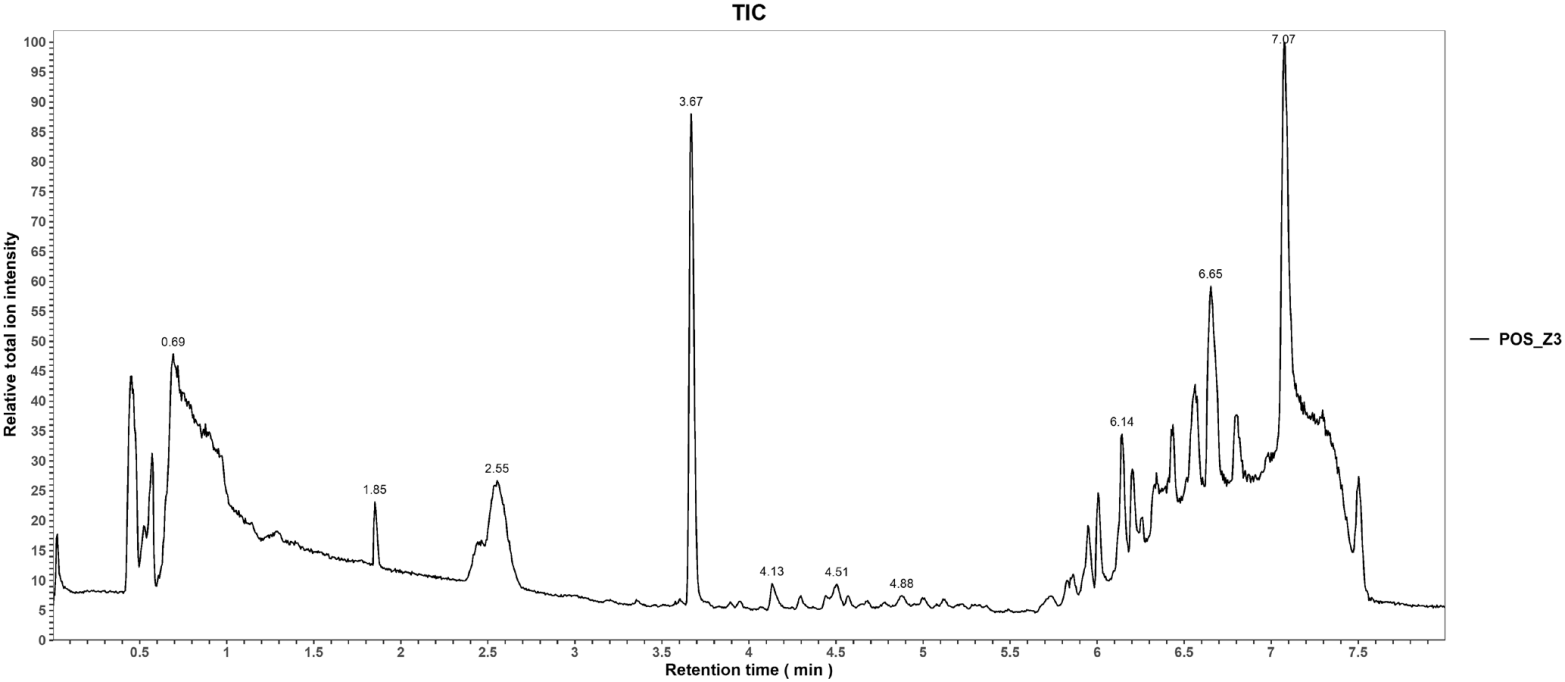
Comparative metabolomic profiling of *Citrus aurantium* honey and blank control (positive ionization mode).

**FIGURE 2:**
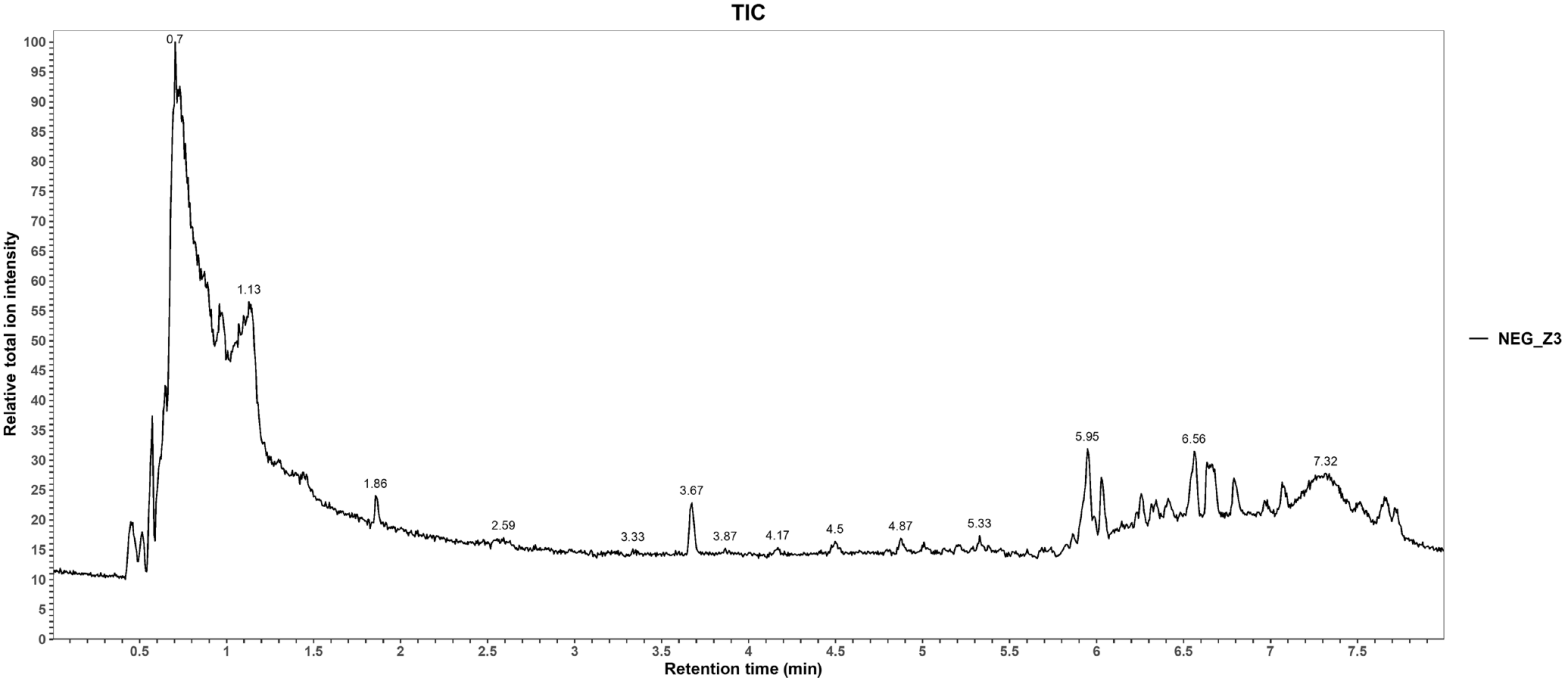
Comparative metabolomic profiling of *Citrus aurantium* honey and blank control (negative ionization mode).

The chemical profile of *C. aurantium* honey was characterized using UHPLC‐Q Exactive HF‐MS. Analysis was performed in both positive and negative ionization modes, resulting in the detection of 2618 distinct chemical components. Among these, six components exhibited exceptional abundance, with signal intensities exceeding 1 × 10^8^. These high‐abundance compounds, ranked in descending order of their mass spectral response, were identified as dioctyl succinate, isomaltose (a panose‐disaccharide), isomaltulose(2‐*O*‐α‐d‐glucopyranosyl‐d‐glucose), glyceraldehyde, and oleamide. Total ion chromatograms (TICs) were acquired for both *C. aurantium* honey and a procedural blank in positive and negative electrospray ionization modes, as shown in Figures 1 and 2, respectively. The complete set of metabolites identified from the honey sample is cataloged in Table 4.

**TABLE 4:** High‐abundance bioactive components in *Citrus aurantium* honey prioritized using spectral intensity.

Serial number	Identification result	UID	Response intensity
1	Dioctyl succinate	P18031	3.10 × 10^9^
2	Panose	P41595	4.07 × 10^8^
3	Kojibiose	P11473	2.41 × 10^8^
4	Glyceraldehyde	P15121	2.22 × 10^8^
5	3‐Deoxyglucosone	Q07869	1.47 × 10^8^
6	Oleamide	P06493	1.17 × 10^8^
7	Raffinose	P21554	9.13 × 10^7^
8	Difructose anhydride III	P04278	9.01 × 10^7^
9	Tricoumaroyl spermidine	P35790	8.83 × 10^7^
10	Gluconic acid	P22303	8.52 × 10^7^

### 3.2 Network pharmacology‐based screening of *C. aurantium* honey targets for gastrointestinal motility

A target screening approach was employed to investigate the potential mechanisms of *C. aurantium* honey in regulating intestinal function. Initial database queries of PubChem and SwissTargetPrediction identified 132 potential bioactive targets (*p* > 0) following deduplication. Concurrently, disease‐related targets were retrieved from GeneCards and OMIM using the search terms “intestinal motility,” “intestinal emptying,” and “intestinal peristalsis,” yielding 12 161; 2819; and 411 targets, respectively.

**FIGURE 3:**
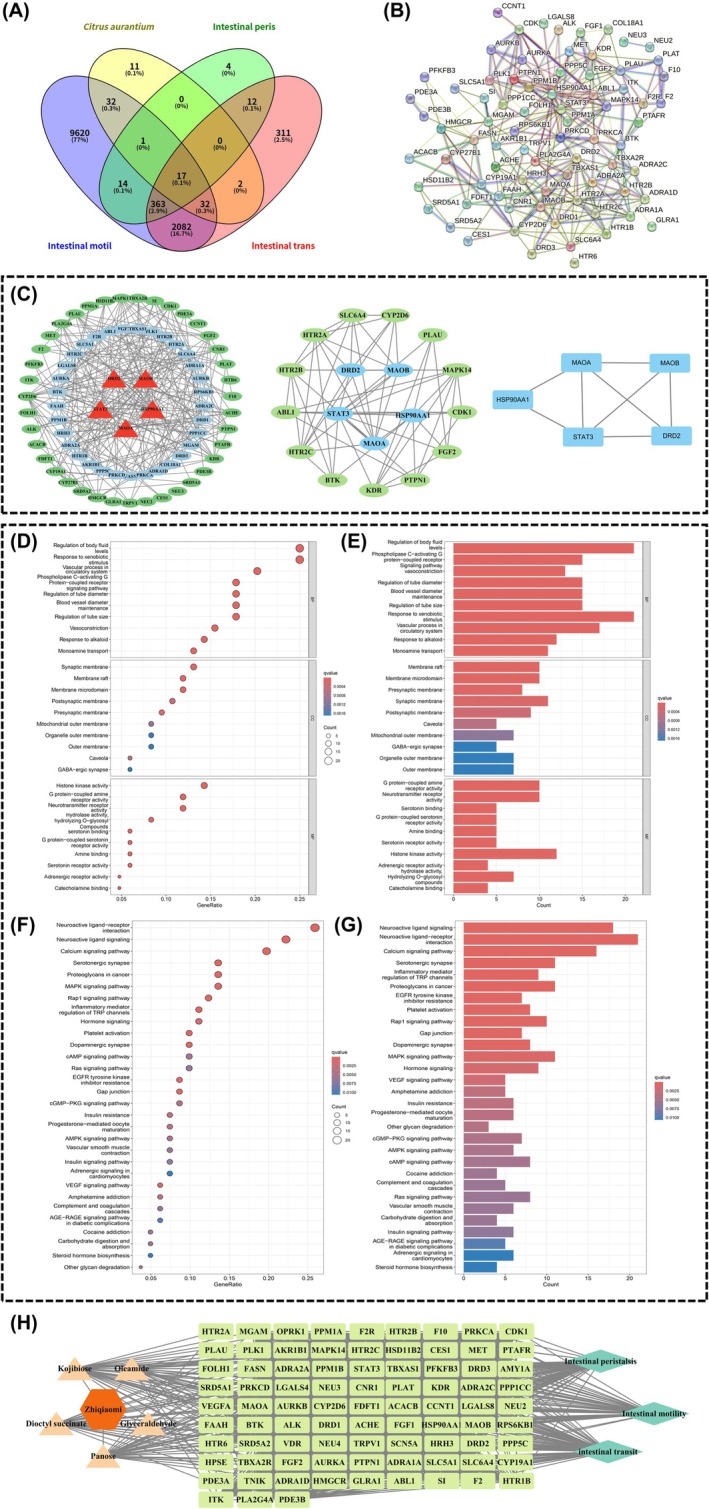
Network pharmacology elucidates the multitarget mechanisms of *Citrus aurantium* honey in regulating gastrointestinal motility. (A) Identification of core targets shared between *C. aurantium* honey bioactives and gastrointestinal motility processes. A Venn diagram shows the intersection of targets from honey constituents and motility‐related pathways, revealing 17 core candidate targets. (B) Protein–protein interaction (PPI) network of the core targets. Nodes represent proteins, and edges represent interactions. The topology reveals densely connected functional modules implicated in motility regulation. (C) Mechanistic network of multilevel interactions. This network illustrates the hierarchical regulatory relationships (e.g., activation, inhibition) among the core targets, from upstream signaling to downstream effectors. (D) Prioritization of key regulatory hubs from the global PPI network. Core hubs (HSP90AA1, MAOA, MAOB, STAT3, and DRD2) were identified from an 83‐node, 287‐edge network through centrality analysis and are highlighted as pivotal mediators. (E, F) Gene Ontology (GO) enrichment analysis of the core targets. (E) Bar plot of the top 10 significantly enriched GO terms. Key enriched biological processes include fluid homeostasis and synaptic signaling; cellular components include membrane microdomains and synapses; molecular functions include GPCR activity and neurotransmitter binding. (F) Bubble plot providing a comprehensive overview of GO enrichment. Bubble size represents the number of genes, and color represents the significance of enrichment. (G, H) Kyoto Encyclopedia of Genes and Genomes (KEGG) pathway enrichment analysis. (G) Schematic map of key enriched KEGG pathways, such as calcium and cGMP‐PKG signaling, highlighting the integration of core targets into these motility‐relevant cascades. (H) Bubble plot of the top 30 significantly enriched KEGG pathways. Critical pathways for gastrointestinal motility, including neuroactive ligand–receptor interaction and vascular smooth muscle contraction, are prominently enriched.

Integration of the intestinal emptying and peristalsis target sets produced 380 unique targets common to both processes. Comparative analysis using the Venny 2.1.0 platform (Figure 3A) revealed 17 overlapping targets between *C. aurantium* honey and the combined intestinal emptying/peristalsis target set, suggesting potential key mediators of its gastrointestinal activity.

### 3.3 Acquisition of PPI network genes underlying *C. aurantium* honey's effects on intestinal emptying and peristalsis

The 17 overlapping targets were analyzed using the STRING database (version 12.0) to construct a PPI network, which was subsequently downloaded in TSV format (Figure 3B). This data file was imported into Cytoscape (version 3.10.3) for network visualization and analysis (Figure 3C). The resulting PPI network consisted of 83 nodes and 287 edges, representing 83 target proteins at the intersection of active compounds and disease‐related genes, along with 287 interaction relationships. Network topology was then assessed using the CytoNCA plugin, which calculated six centrality metrics: betweenness (BC), closeness (CC), degree (DC), local average connectivity (LAC), neighborhood connectivity (NC), and eigenvector (EC). Targets exceeding the median value for all six metrics were selected using R language, generating 18 subnetworks. A second round of screening with identical criteria identified five core targets: HSP90AA1, MAOA, MAOB, STAT3, and DRD2.

### 3.4 GO enrichment analysis reveals key biological processes in gastrointestinal function regulation

GO enrichment analysis identified 1182 terms in BP, 31 in cellular component (CC), and 120 in molecular function (MF).^20^ The top 10 most significantly enriched terms from each category, ranked by *p*‐value, were selected to generate a bar chart and a bubble chart (Figure 3D,E). The results demonstrate that the intersecting genes are primarily associated with BP terms, including fluid homeostasis, the phospholipase C‐activating G‐protein–coupled receptor (GPCR) signaling pathway, the regulation of vasoconstriction and vascular diameter, and the response to xenobiotic stimuli. Mechanistically, phospholipase Cβ (PLCβ) is activated by GPCRs and catalyzes the hydrolysis of phosphatidylinositol 4,5‐bisphosphate (PIP_2_) into inositol 1,4,5‐trisphosphate (IP₃) and diacylglycerol (DAG). This signaling cascade constitutes a key step in the contraction of gastrointestinal smooth muscle cells.^20^ Concurrently, fluid homeostasis bidirectionally modulates peristaltic rhythms via the action of gastrointestinal hormones.^21^

Cellular component enrichment analysis demonstrated significant localization of the intersecting genes to several membrane‐associated structures, including membrane rafts, membrane microdomains, presynaptic and postsynaptic membranes, mitochondrial outer membranes, and caveolae. Membrane rafts serve as crucial platforms for organizing neurotransmitter receptors and their downstream signaling components, thereby ensuring efficient synaptic transmission.^22^ Concurrently, mitochondrial outer membranes facilitate essential energy production required for sustained smooth muscle contraction.^23^ Additionally, molecular function analysis identified serotonin‐binding activity as a direct regulator of gastrointestinal motility frequency, working in concert with these structural components to maintain digestive rhythm.^24^

Molecular function enrichment analysis identified significant enrichment of the intersecting genes in G protein–coupled amine receptor activity, neurotransmitter receptor binding (including specific serotonin binding), histone kinase activity, and catecholamine binding. These molecular functions coordinate complementary signaling roles: G protein–coupled receptors and neurotransmitter receptors primarily mediate extracellular signal reception, whereas histone kinase activity and catecholamine binding contribute to intracellular signal transduction and regulation.^25^, ^26^, ^27^, ^28^ Together, these results establish a molecular framework through which the intersecting genes may maintain homeostatic control of intestinal motility, integrating both extracellular signal detection and intracellular signaling pathways.

### 3.5 KEGG pathway enrichment analysis

KEGG pathway enrichment analysis identified 50 significantly enriched metabolic pathways. The top 20 pathways are detailed in Table 5, whereas the 30 most significant pathways (based on *p*‐value) are visualized in a bubble chart and a bar chart (Figure 3F,G). In this representation, bubble size corresponds to the number of genes enriched in each pathway, with larger bubbles indicating higher gene counts. Analysis revealed that the intersecting genes are primarily associated with key signaling pathways, including calcium signaling pathway, vascular smooth muscle contraction, cGMP‐PKG signaling pathway, cAMP signaling pathway, and neuroactive ligand–receptor interaction.

**TABLE 5:** Top 20 KEGG pathways enriched in the intersecting gene set.

Pathway ID	Pathway name	*p*	Number of genes
hsa04080	Neuroactive ligand–receptor interaction	2.62 × 10^−12^	21
hsa04082	Neuroactive ligand signaling	4.52 × 10^−14^	18
hsa04020	Calcium signaling pathway	3.35 × 10^−10^	16
hsa04726	Serotonergic synapse	3.23 × 10^−09^	11
hsa05205	Proteoglycans in cancer	1.19 × 10^−6^	11
hsa04010	MAPK signaling pathway	4.67 × 10^−5^	11
hsa04015	Rap1 signaling pathway	1.24 × 10^−5^	10
hsa04750	Inflammatory mediator regulation of TRP channels	1.49 × 10^−7^	9
hsa04081	Hormone signaling	1.01 × 10^−4^	9
hsa04151	PI3K‐Akt signaling pathway	3.72 × 10^−3^	9
hsa04611	Platelet activation	1.15 × 10^−5^	8
hsa04728	Dopaminergic synapse	1.62 × 10^−5^	8
hsa04024	cAMP signaling pathway	6.85 × 10^−4^	8
hsa04014	Ras signaling pathway	9.61 × 10^−4^	8
hsa01521	EGFR tyrosine kinase inhibitor resistance	5.08 × 10^−6^	7
hsa04540	Gap junction	1.29 × 10^−5^	7
hsa04022	cGMP‐PKG signaling pathway	5.36 × 10^−4^	7
hsa05207	Chemical carcinogenesis—receptor activation	2.54 × 10^−3^	7
hsa04931	Insulin resistance	3.33 × 10^−4^	6
hsa04914	Progesterone‐mediated oocyte maturation	3.67 × 10^−4^	6

The calcium signaling pathway orchestrates contractile responses in intestinal smooth muscle cells through modulation of intracellular Ca^2+^ concentrations, thereby serving as the primary mechanism responsible for sustaining basal intestinal motility.^29^

The cGMP‐PKG signaling pathway exerts anti‐inflammatory effects and preserves intestinal barrier integrity by suppressing mTORC1 protein expression, thereby ameliorating colitis symptoms.^30^

### 3.6 Construction of an integrated bioactive‐target‐intestinal motility network for *C. aurantium* honey

To elucidate the complex interactions between *C. aurantium* honey and intestinal motility regulation, we constructed an integrated network using Cytoscape (version 3.10.3). This “*C. aurantium* honey‐constituents‐targets‐pathophysiology” network (Figure 3H) systematically integrates four key elements: *C. aurantium* honey (represented by regular hexagons), its bioactive constituents (triangles), drug–disease intersecting targets (rectangles), and intestinal motility phenotypes (rhombi), including emptying, propulsion, and peristalsis. This integrated representation demonstrates the multilevel pharmacological relationships through which *C. aurantium* honey constituents potentially modulate gastrointestinal function, providing a comprehensive framework for understanding its mechanistic basis in intestinal motility regulation.

### 3.7 Verification of core target interactions via molecular docking

Based on the preceding research results, five major bioactive constituents of kumquat honey were selected for molecular docking analysis: dioctyl succinate, panose (also known as panose disaccharide), isomaltulose (2‐glucopyranosyl‐α‐d‐glucopyranoside), glyceraldehyde, and oleamide. These compounds were docked with five core targets identified through network pharmacology screening: MAOA, MAOB, DRD2, STAT3, and HSP90AA1. The binding conformations between target proteins and ligands were optimized and visualized using PyMOL software. Analysis revealed strong molecular interactions, including hydrogen bonding, hydrophobic interactions, electrostatic forces, and van der Waals forces. The binding affinity of these complexes was quantified by binding energy.^31^

**FIGURE 4:**
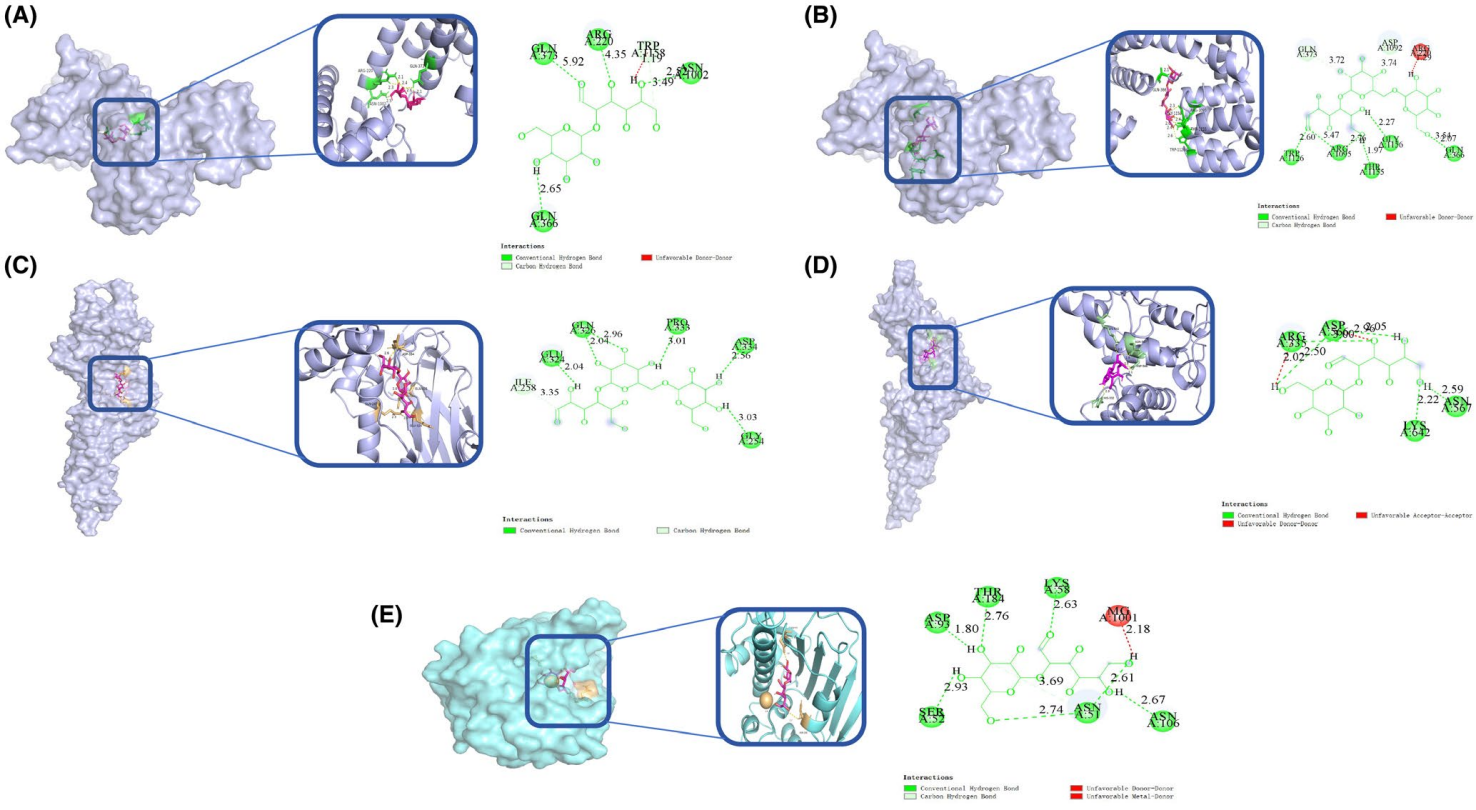
Molecular docking reveals multitarget mechanisms of *Citrus aurantium* peel honey in regulating gastrointestinal motility. Molecular docking reveals high‐affinity binding of *C. aurantium* honey bioactives to key targets in gastrointestinal regulation. (A) Binding pose of Kojibiose within the ligand‐binding pocket of the dopamine receptor DRD2 (binding energy = −5.4 kcal/mol). The complex is stabilized by specific hydrogen bonds (green dashes) and hydrophobic interactions with key residues (green sticks), suggesting a potential role in modulating dopaminergic pathways that influence gut motility. (B) Panose engages a primary binding site on the transcription factor DRD2 (binding energy = −6.1 kcal/mol). Interactions with residues such as GLN and ARG indicate direct binding, supporting a mechanism for the suppression of STAT3‐mediated pro‐inflammatory signaling. (C) Panose exhibits superior binding affinity for a distinct site on STAT3 (binding energy = −6.5 kcal/mol). Its unique interaction network with residues, including ILE and SER, suggests a synergistic role with Kojibiose in the potent inhibition of STAT3 activity. (D) Kojibiose occupies a secondary, allosteric binding site on STAT3 (binding energy = −6.1 kcal/mol). This spatially distinct binding mode from (B) reveals a polypharmacological mechanism whereby a single compound can target multiple sites on the same protein to enhance efficacy. (E) Kojibiose forms a stable complex with the ATPase domain of the molecular chaperone HSP90AA1 (binding energy = −5.9 kcal/mol). A hydrogen‐bonding network with ASN and THR residues suggests that Kojibiose may modulate protein homeostasis and client protein stability in gastrointestinal tissues.

Binding affinities were evaluated based on calculated binding energies: values below −5.0 kcal/mol indicate favorable ligand–receptor interactions, whereas energies below −7.0 kcal/mol represent strong binding capability.^32^ Molecular docking results showed that two key constituents of kumquat honey—panose and isomaltulose—could effectively bind to three core target proteins with significant affinity: DRD2 (Figure 4A,B), STAT3 (Figure 4C,D), and HSP90AA1 (Figure 4E). The calculated binding energies ranged from −5.4 to −6.2 kcal/mol, all below the established threshold of −5.0 kcal/mol for spontaneous molecular interactions. Structural visualization and interaction analysis of the obtained complexes were conducted using PyMOL and Discovery Studio, revealing stable binding conformations and specific molecular interactions that underpin the observed binding affinities.

Molecular docking analyses predict high‐affinity interactions between bioactive compounds from *C. aurantium* peel honey and key protein targets involved in gastrointestinal motility, supporting a polypharmacological mode of action.

### 3.8 Effects of *C. aurantium* honey on intestinal motility in *D. melanogaster*

#### 3.8.1 Effect of *C. aurantium* honey on fecal output in *D. melanogaster*

**FIGURE 5:**
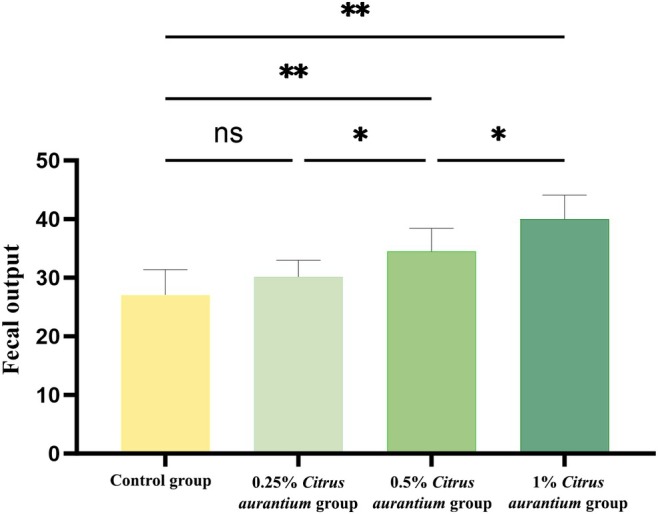
*Citrus aurantium* honey stimulates defecation in a dose‐dependent manner in *Drosophila*. *p < 0.05; **p < 0.01.

Analysis (Figure 5) of fecal output in *D. melanogaster* revealed a concentration‐dependent response to *C. aurantium* honey supplementation. The defecation assay in *D. melanogaster* revealed that flies fed a 1% concentration of *C. aurantium* honey exhibited a significantly higher excretion rate compared to the control group. This effect is likely attributable to the enhancement of intestinal motility induced by the honey. Moreover, fecal output showed a progressive increase with ascending concentrations of *C. aurantium* honey (0.25%, 0.5%, and 1%), demonstrating a clear concentration‐dependent response. These results indicate that within the experimental concentration range, higher concentrations of *C. aurantium* honey may exert a stronger promotive effect on defecation.

#### 3.8.2 *C. aurantium* honey potently stimulates feeding in *D. melanogaster* in a dose‐dependent manner

**FIGURE 6:**
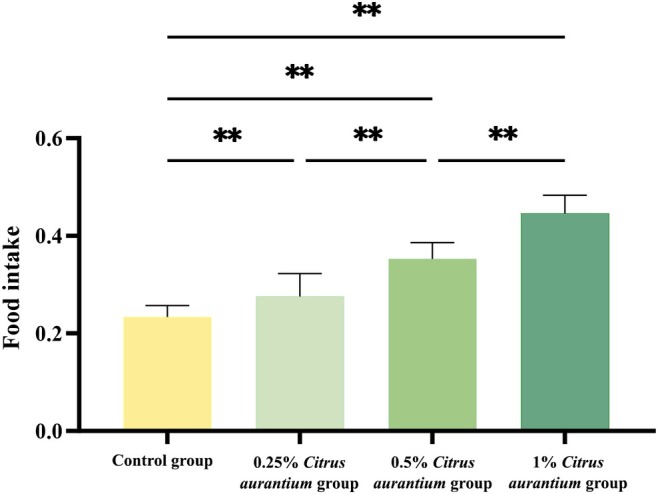
*Citrus aurantium* honey promotes feeding behavior in a dose‐dependent manner. **p < 0.01.

Dietary supplementation with *C. aurantium* honey robustly enhanced food intake in *D. melanogaster* (Figure 6). Flies consuming diets supplemented with 0.5% and 1% honey exhibited a significant increase in consumption compared to the control group (*p* < 0.01), with a more modest effect observed at 0.25%. This dose‐dependent stimulation of feeding behavior, coupled with the concurrent enhancement of intestinal motility, indicates that *C. aurantium* honey acts as a multifaceted regulator of digestive physiology, potentially through coordinated mechanisms that promote both ingestion and gastrointestinal transit.

#### 3.8.3 Effect of *C. aurantium* honey on climbing ability in *D. melanogaster*

**FIGURE 7:**
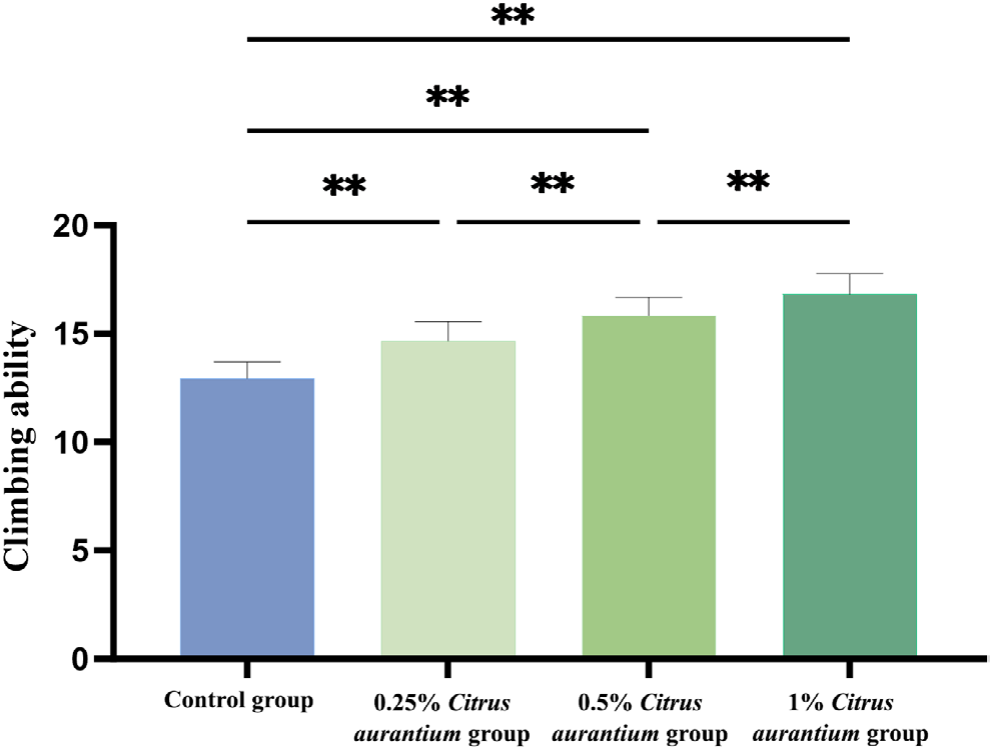
*Citrus aurantium* honey enhances climbing ability of *Drosophila melanogaster* in a concentration‐dependent manner. **p < 0.01.

In the model organism *D. melanogaster*, the onset of intestinal pathology is frequently associated with a decline in locomotor performance.^33^ We evaluated climbing ability, a key indicator of locomotor function, in *D. melanogaster* following dietary supplementation with *C. aurantium* honey. The results revealed significant, dose‐dependent effects compared to the untreated control group (Figure 7). A significant, dose‐dependent enhancement in climbing performance was observed across all treatment groups (*p* < 0.01). The improvement was most substantial at the 1% concentration, intermediate at 0.5%, and modest yet significant at 0.25%. This establishes *C. aurantium* honey as a potent stimulator of locomotor function in a dose‐dependent manner.

#### 3.8.4 *C. aurantium* honey dose‐dependently accelerates intestinal transit in *D. melanogaster*

**FIGURE 8:**
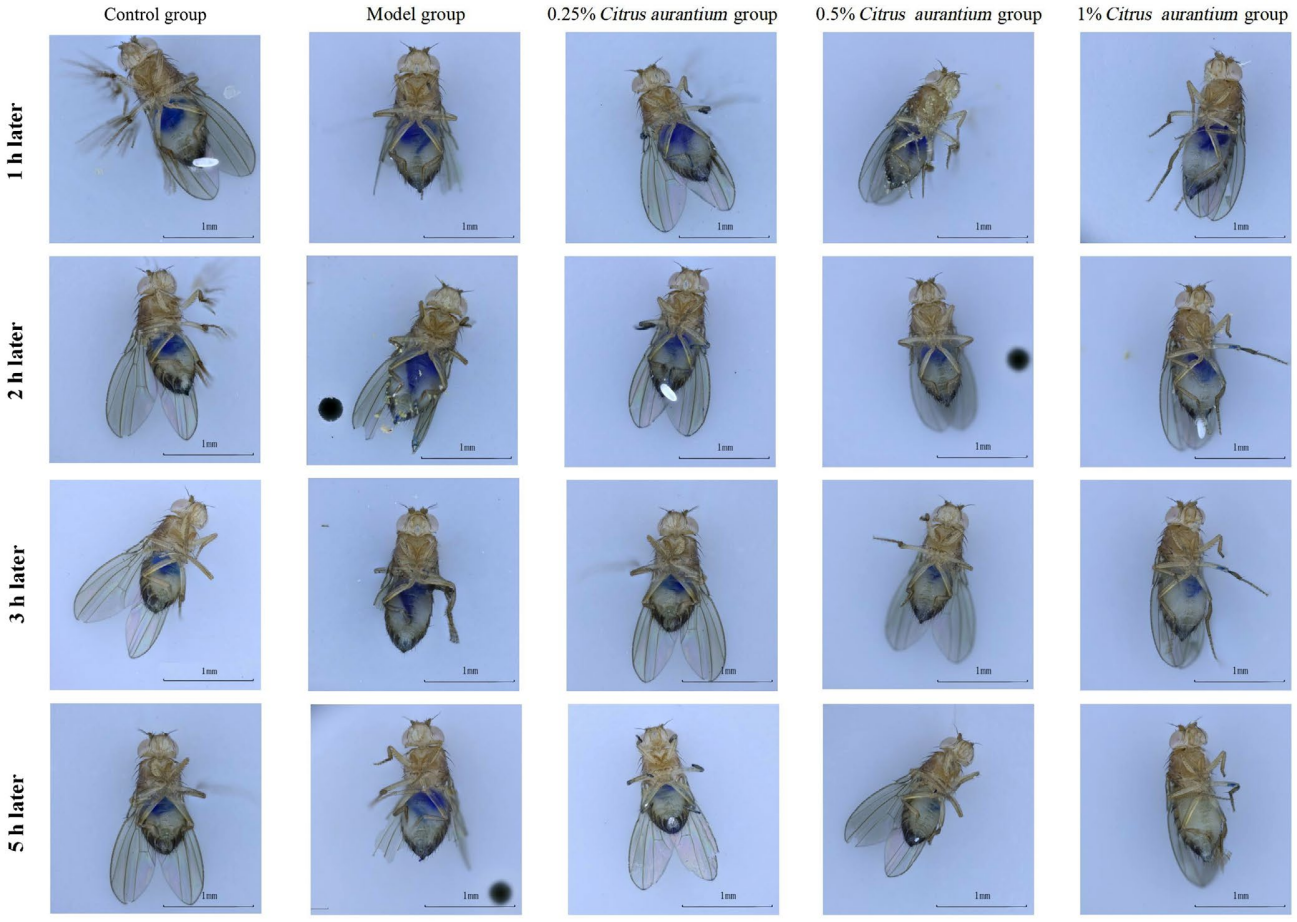
*Citrus aurantium* honey accelerates intestinal transit in a dose‐dependent manner.

The clearance of a fluorescent marker from the *Drosophila* gut was quantified to assess intestinal transit dynamics following *C. aurantium* honey treatment (Figure 8). At the 8‐h time point, a striking dose‐dependent response was observed. The gut of flies treated with 1% honey was completely cleared of the marker. Although residual fluorescence was present at lower concentrations, its level in the 0.5% group was substantially reduced compared to the 0.25% group. Critically, all honey‐treated groups exhibited significantly faster clearance than the blank control. These results demonstrate that *C. aurantium* honey potently and dose‐dependently enhances intestinal transit.

#### 3.8.5 *C. aurantium* honey protects against LPS‐induced intestinal injury in *D. melanogaster*

**FIGURE 9:**
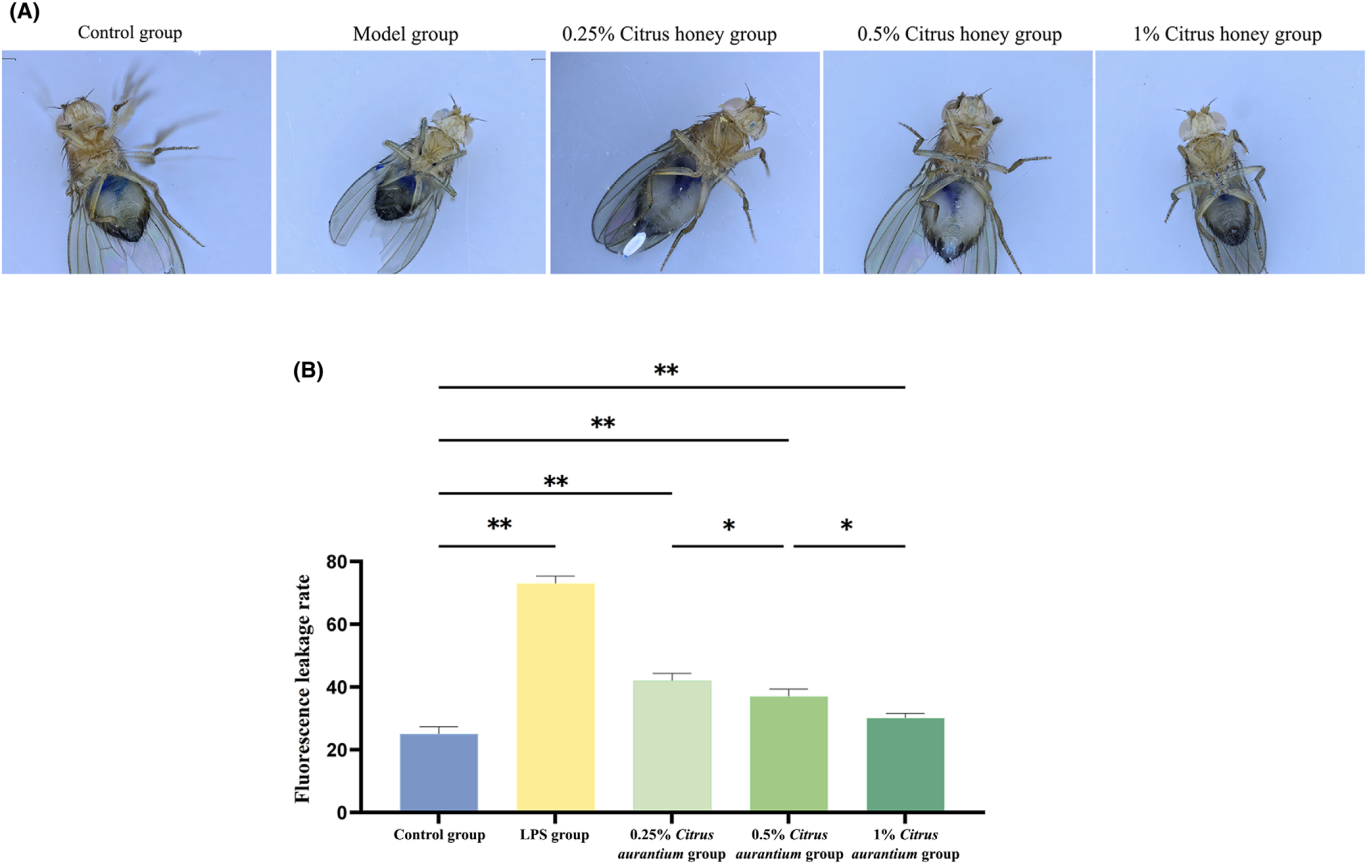
*Citrus aurantium* honey ameliorates lipopolysaccharide (LPS)‐induced intestinal barrier damage in *Drosophila melanogaster*. *p < 0.05, **p < 0.01. (A) Representative images of *Drosophila* from the following groups: (1) untreated control, (2) LPS‐induced injury model, and LPS‐injured flies fed with (3) 0.25%, (4) 0.5%, or (5) 1% *C. aurantium* honey. Honey administration restores intestinal integrity in a dose‐dependent manner. Scale bars = 1 mm. (B) Quantitative assessment of intestinal barrier function using fluorescence leakage assay. Data are presented as mean ± standard error of the mean (SEM). **p < 0.01 versus the LPS model group (one‐way analysis of variance [ANOVA]). LPS challenge induces a profound increase in gut permeability, which is significantly and dose‐dependently rescued by *C. aurantium* honey treatment.

Representative images of intestinal tissue revealed that *C. aurantium* honey ameliorated the histopathological damage induced by LPS in *Drosophila* (Figure 9A). Concordant with these morphological observations, functional assessment of intestinal barrier integrity showed a significantly elevated leakage rate in the model group compared to the blank control (Figure 9B). Conversely, all *C. aurantium* honey treatment groups exhibited a substantial reduction in this leakage rate. Collectively, these morphological and functional data demonstrate that *C. aurantium* honey confers a significant protective effect against LPS‐induced intestinal injury in *Drosophila*.

#### 3.8.6 *C. aurantium* honey attenuates LPS‐induced intestinal oxidative stress in *D. melanogaster*

**FIGURE 10:**
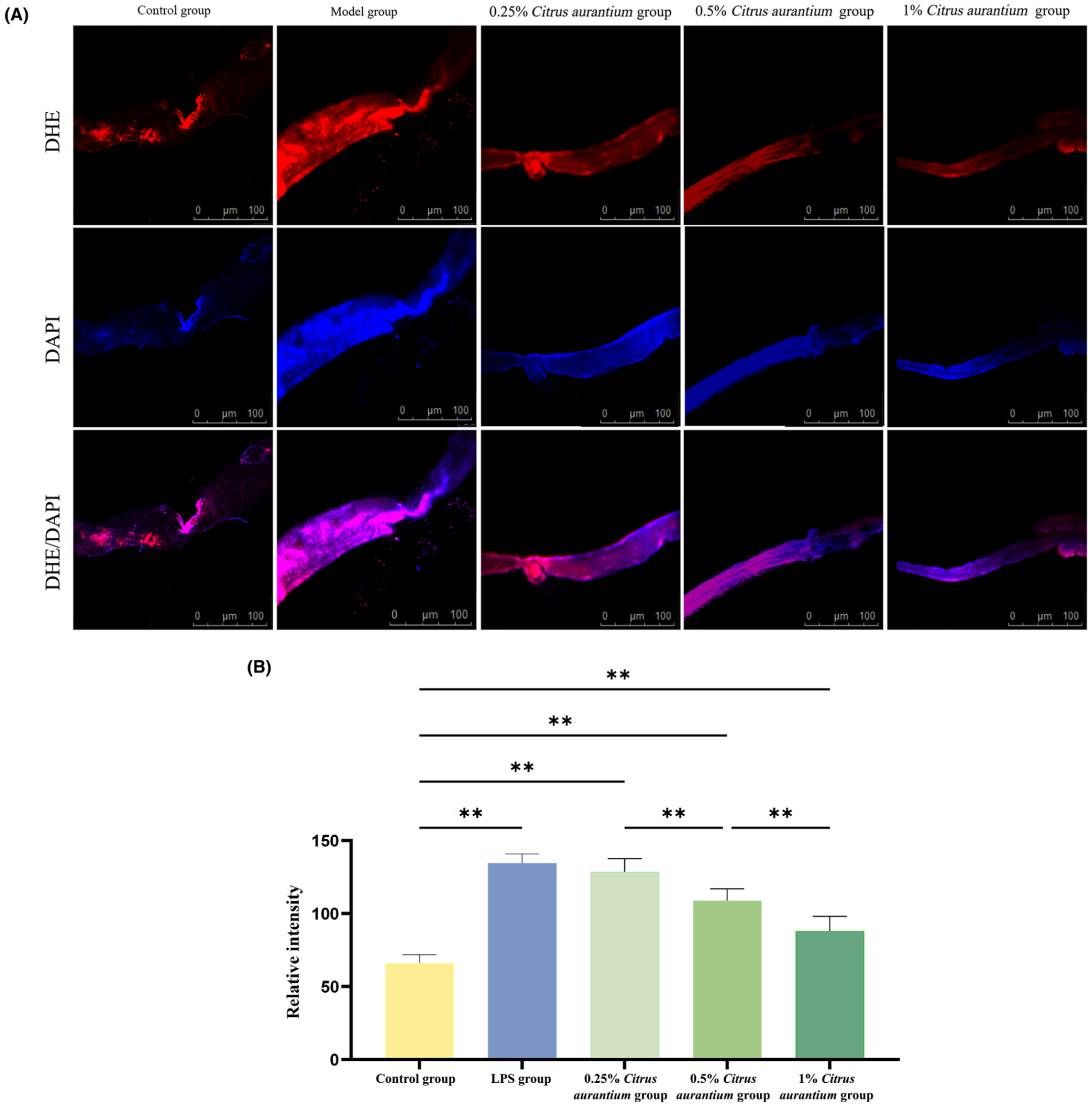
*Citrus aurantium* honey attenuates LPS‐induced intestinal oxidative stress in *Drosophila* through dose‐dependent ROS suppression. (A) Representative fluorescence micrographs of *Drosophila* midguts stained with dihydroethidium DHE (red, reactive oxygen species [ROS] indicator) and DAPI (blue, nuclei). From left: Control (untreated), lipopolysaccharide (LPS)‐induced injury model, and LPS‐injured flies supplemented with increasing concentrations (0.25%, 0.5%, 1%) of *C. aurantium* honey. LPS challenge triggers substantial ROS accumulation, whereas honey treatment dose‐dependently restores redox homeostasis. Scale bars = 100 μm. (B) Quantitative analysis of intestinal ROS levels measured by DHE fluorescence intensity. Data represent mean ± standard error of the mean (SEM) from three independent experiments (*n* ≥ 15 intestines per group). **p < 0.01 versus LPS model group (one‐way analysis of variance [ANOVA] with Tukey's post‐hoc test). *C. aurantium* honey significantly abrogates LPS‐induced oxidative stress in a concentration‐dependent manner.

Intestinal oxidative stress, marked by elevated ROS, is a hallmark of inflammatory gut pathology. As demonstrated in Figure 10A,B, LPS challenge significantly increased intestinal ROS levels in *Drosophila* compared to the blank control group. In contrast, coadministration of *C. aurantium* honey with LPS substantially suppressed this oxidative burst. The attenuation of intestinal ROS was further corroborated by a marked reduction in DHE fluorescence intensity in honey‐treated groups. Notably, this antioxidant activity was concentration‐dependent: higher doses of *C. aurantium* honey elicited progressively stronger suppression of ROS and greater amelioration of intestinal inflammation. Taken together, these findings establish *C. aurantium* honey as an effective natural intervention against LPS‐triggered oxidative stress and inflammation in the *Drosophila* intestine, highlighting its dual role as an antioxidant and anti‐inflammatory agent.

#### 3.8.7 *C. aurantium* honey modulates TORC1‐4E‐BP in *D. melanogaste*

**FIGURE 11:**
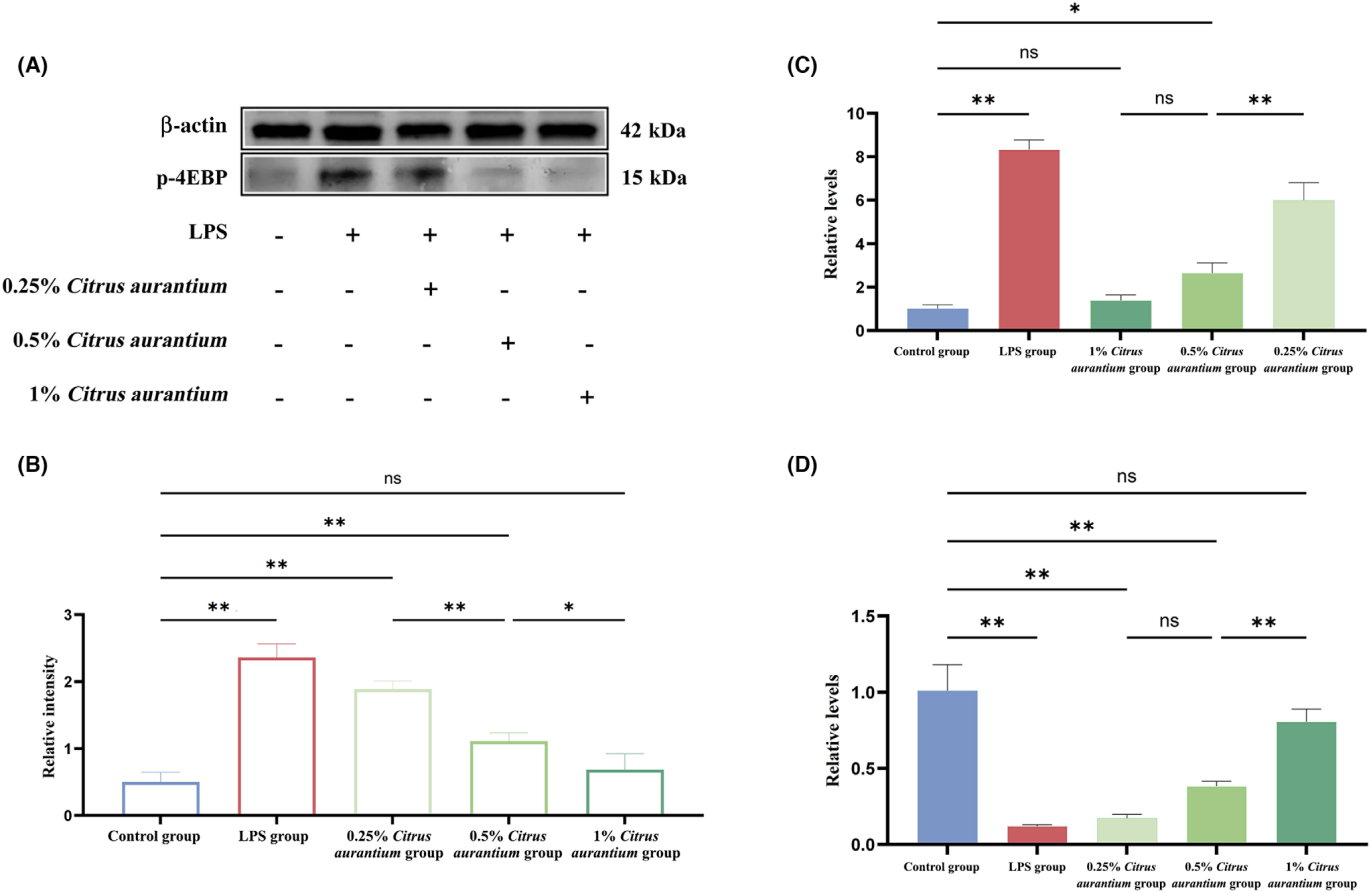
*Citrus aurantium* honey alleviates LPS‐induced intestinal dysfunction in *Drosophila* via TORC1 signaling modulation. (A) Representative Western blot showing phosphorylated 4E‐BP (p‐4E‐BP, 15 kDa) and β‐actin (42 kDa) protein levels in *Drosophila* intestines across experimental groups. (B) Quantitative analysis of p‐4E‐BP protein levels normalized to β‐actin. Data represent mean ± standard error of the mean (SEM). **p < 0.01 versus LPS group; ns, not significant (one‐way analysis of variance [ANOVA]). (C) Relative messenger RNA (mRNA) expression of Thor, a downstream component of TORC1 signaling. Data represent mean ± SEM. **p < 0.01, *p < 0.05 versus LPS group; ns, not significant. (D) Relative mRNA expression of Nprl, a negative regulator of TORC1. Data represent mean ± SEM. **p < 0.01 versus control group (one‐way ANOVA). *C. aurantium* honey treatment counteracts LPS‐induced TORC1 hyperactivation through dose‐dependent reduction in p‐4E‐BP phosphorylation, suppression of Thor expression, and restoration of Nprl levels, collectively demonstrating its efficacy in restoring intestinal homeostasis.

Western blot analysis of phosphorylated 4E‐BP (p‐4EBP) in *Drosophila* intestines. LPS challenge increased p‐4EBP levels, indicating TORC1 pathway activation, whereas *C. aurantium* honey treatment (0.25%–1%) dose‐dependently reduced p‐4EBP. β‐Actin served as a loading control (Figure 11A,B).

#### 3.8.8 *C. aurantium* honey suppresses LPS‐induced upregulation of TOR pathway genes in *D. melanogaster*

As demonstrated in Figure 11C, *C. aurantium* honey dose‐dependently suppressed LPS‐induced Thorl expression in *D. melanogaster*. Under basal conditions, Thorl transcript levels remained low in untreated controls, whereas LPS challenge triggered a significant upregulation (*p* < 0.01). All *C. aurantium* honey treatment groups showed marked attenuation of this LPS‐induced expression, with higher honey concentrations eliciting stronger suppression.

As shown in Figure 11D, *C. aurantium* honey dose‐dependently rescued the LPS‐induced suppression of Nprl expression in *D. melanogaster*. Under basal conditions, Nprl expression remained stable, whereas LPS challenge significantly downregulated Nprl transcript levels compared to the control group (*p* < 0.01).

Treatment with *C. aurantium* honey restored Nprl expression in a concentration‐dependent manner. The 1% honey group completely reversed LPS‐induced suppression (*p* < 0.01), whereas the 0.5% honey group showed significant but partial recovery (*p* < 0.01). The 0.25% honey group showed no statistically significant difference compared to the LPS‐treated group (ns, *p* > 0.05).

## 4 DISCUSSION

Chemical profiling of *C. aurantium* honey using UHPLC‐Q Exactive HF‐MS revealed several bioactive constituents, including diocyl succinate, panoside (panose), kojibiose, 2‐glucosyl‐α‐glucoside, glyceraldehyde, and oleamide. These identified compounds establish a molecular foundation for understanding the mechanistic basis of its intestinal motility‐regulating effects.

Through network pharmacology analysis, we identified 17 potential targets associated with intestinal emptying and motility. Functional enrichment analysis demonstrated significant involvement in calcium signaling, cGMP‐PKG, and cAMP pathways, as well as neuroactive ligand–receptor interactions. Particularly relevant is the established role of dihydropyridine‐sensitive Ca^2+^ channels (Cav1.2) in mediating intestinal contraction, suggesting that calcium signaling modulation may represent a primary mechanism through which *C. aurantium* honey influences gastrointestinal function.

Collectively, these findings delineate a multicomponent, multitarget mechanism by which *C. aurantium* honey regulates intestinal motility, with calcium signaling positioned as a central hub within this regulatory network. Rather than operating in isolation, this pathway interfaces with the integrated neurohormonal and mechanosensitive framework that governs gastrointestinal peristalsis,^34^, ^35^ placing calcium signaling at a nodal intersection of multiple physiological inputs. The clinical relevance of this mechanism is underscored by its convergence with the pathological architecture of gastrointestinal dysmotility—conditions that not only present as primary disorders but frequently arise as postoperative complications or comorbidities in diseases such as IBD and Parkinson's disease (PD).^36^ Autonomic dysregulation and psychological stress are known to impair motility via neurotransmitter imbalance^37^; notably, the calcium signaling pathway enriched in our study overlaps substantially with these pathological axes. This alignment between molecular enrichment and clinical pathological characteristics not only provides a mechanistic rationale for the prokinetic effects of *C. aurantium* honey but also positions calcium signaling as a candidate intervention node in motility disorders of diverse etiologies.

Intestinal motility is governed by an integrated signaling network in which the cAMP, cGMP‐PKG, and TOR pathways converge to coordinate neural transmission, smooth muscle contraction, and metabolic adaptation. Within this framework, the cGMP‐PKG axis suppresses inflammation and preserves motor function via PKG‐dependent regulation of contractile machinery and mTORC1 activity.^38^, ^39^, ^40^ However, under LPS‐induced inflammatory stress, TORC1 signaling exhibits context‐dependent duality: although its activation mediates protective lipid remodeling and epithelial repair, sustained hyperactivation in response to chronic inflammation exacerbates dysmotility.^41^ Our findings identify the Thor/Nprl2‐TORC1 axis as a previously unrecognized regulatory node within this network. By linking nutrient sensing to TORC1 activity, this mechanism operates upstream of the cGMP‐PKG–mTORC1 cascade and provides a targetable checkpoint for modulating motility under inflammatory conditions. This convergence with the pathophysiological architecture of IBD and related motility disorders positions Thor/Nprl2‐TORC1 signaling not merely as a downstream responder but as a decisive rheostat in the transition from protective adaptation to pathological dysmotility.

Concurrently, the enrichment of specific membrane microdomains—including membrane rafts and synaptic membranes—establishes a structural foundation for neurotransmission‐mediated intestinal motility signaling.^42^, ^43^ This molecular pathway likely constitutes the fundamental mechanism through which *C. aurantium* honey mediates its dual prokinetic and anti‐inflammatory therapeutic properties.

Intestinal motility serves as a critical determinant of normal bowel function, and its dysregulation may lead to various clinical manifestations, including defecation abnormalities and appetite dysregulation.^44^ Our findings further underscore the need to elucidate the mechanism of action of *C. aurantium* honey. To validate the proposed mechanism, we systematically evaluated its regulatory effects on intestinal motility using a *Drosophila* model designed to assess three dimensions: intestinal transit, feeding behavior, and overall physiological status. Regarding direct motility indicators, the high‐dose *C. aurantium* honey group showed significantly higher defecation rates and food intake compared to the blank control group. This indicates that *C. aurantium* honey effectively accelerates intestinal content transit and may be associated with activation of a feedback loop between enhanced intestinal emptying and feeding behavior—where rapid intestinal clearance reduces intraluminal pressure, thereby promoting feeding. Furthermore, climbing ability—an indirect measure of gut health reflecting overall physiological status—significantly improved with increasing concentrations of *C. aurantium* honey. This suggests that improved intestinal motility concurrently restores the flies' locomotor capacity, further substantiating the positive regulatory role of *C. aurantium* honey in gastrointestinal physiological function.

The integrity of the intestinal barrier and controlled inflammatory status represent critical determinants of intestinal homeostasis. As a well‐characterized inflammatory inducer, LPS compromises intestinal barrier function and elicits oxidative stress responses, thereby elevating ROS levels and ultimately contributing to intestinal dysfunction.^45^, ^46^, ^47^ Results showed a significantly elevated intestinal leakage rate in the LPS‐induced intestinal injury model group compared to the control group. In contrast, all *C. aurantium* honey treatment groups exhibited markedly reduced leakage rates, indicating its efficacy in alleviating LPS‐mediated intestinal barrier disruption and exerting barrier‐protective effects. Regarding inflammatory suppression, LPS challenge significantly increased intestinal ROS levels—an established inflammatory activity marker—in *D. melanogaster*. However, *C. aurantium* honey supplementation dose‐dependently reduced both intestinal ROS levels and corresponding fluorescence intensity. These findings suggest that *C. aurantium* honey protects intestinal barrier function by inhibiting LPS‐induced inflammation and mitigating intestinal mucosal damage, as evidenced by reduced leakage rates. Simultaneously, intestinal emptying efficiency improved progressively with increasing honey concentrations. These results indicate that *C. aurantium* honey exerts anti‐inflammatory and antioxidant effects through ROS suppression, thereby establishing a pathological basis for improved intestinal motility and forming a logical mechanistic loop: anti‐inflammation → barrier protection → motility enhancement. Evidence indicates that elevated TORC1 protein activity directly induces delayed gastrointestinal emptying and impaired motility through enhanced phosphorylation of its downstream effector, 4E‐BP.^48^ In this study, LPS‐induced intestinal inflammation promoted further activation of the TOR signaling pathway, consequently aggravating intestinal motility dysfunction. Molecular investigation demonstrated that *C. aurantium* honey treatment significantly reduced the phosphorylation of 4E‐BP, a key downstream effector of TORC1, as evidenced by Western blot analysis. This finding indicates the potential of *C. aurantium* honey to suppress TORC1 overactivation. Further confirmation through PCR analysis revealed that *C. aurantium* honey downregulated the expression of the 4E‐BP gene (Thor) while upregulating its antagonistic gene nprl. These transcriptional alterations were consistent with the observed changes in protein phosphorylation. Together with the previously described phenotypic improvements, these molecular insights establish a coherent mechanistic basis for *C. aurantium* honey's mode of action: by attenuating 4E‐BP phosphorylation, it inhibits LPS‐induced TORC1 signaling hyperactivation, thereby ameliorating intestinal motility disorders through molecular pathway regulation.

Compared to traditional *C. aurantium* herbal medicine, *C. aurantium* honey demonstrates superior efficacy in both gastrointestinal motility regulation and anti‐inflammatory activity, thereby lending substantial support to its potential application in related disease interventions.

The phytochemical composition of *C. aurantium* primarily comprises alkaloids (e.g., synephrine) and flavonoids (e.g., hesperidin), exhibiting notable target specificity. In contrast, *C. aurantium* honey presents a far more complex functional profile, with 2618 major constituents identified—such as dioctyl succinate, panose, kojibiose, 2‐gluco‐α‐glucoside, glyceraldehyde, and oleamide. Such complexity enables the honey to mediate its effects through structural modulation of the gut microbiota. This mode of action is grounded in the well‐established adaptability of the gut microbiome, which evolves throughout life in response to dietary patterns, lifestyle factors, hormonal fluctuations, and immune activity.^49^ Thus, the multicomponent system of *C. aurantium* honey influences gut health primarily by engaging these adaptable, microbiota‐associated pathways. In regulating gastrointestinal motility, *C. aurantium* acts by inhibiting extracellular Ca^2+^ influx and modulating intracellular Ca^2+^ release, thereby reducing smooth muscle contractile tension and producing a relaxant effect. This relaxant effect partially depends on the endothelial NO‐sGC signaling pathway while also involving inhibition of nonselective cation channels and RyR‐mediated Ca^2+^ release.^50^ *C. aurantium* honey achieves comprehensive regulation through multicomponent synergy—oligosaccharides serving as prebiotics can be fermented by beneficial bacteria such as *Bifidobacterium* and *Lactobacillus*, significantly elevating short‐chain fatty acid (SCFA) levels. These SCFAs not only provide the primary energy source for colonic epithelial cells but also maintain intestinal barrier integrity through multiple mechanisms: lowering intestinal pH to inhibit pathogenic bacterial growth, promoting mucus secretion, and enhancing tight junction protein expression.^51^ Oleamide acts as a positive allosteric modulator of 5‐HT_2_A and 5‐HT_2_C receptors, potentiating 5‐HT–evoked signal transduction and thereby enhancing neurotransmitter release. In contrast, at 5‐HT₇ receptors, it functions as a negative allosteric modulator that regulates cAMP signaling and consequently influences neuronal excitability. Collectively, these mechanisms demonstrate that oleamide modulates intestinal neural reflexes and peristaltic rhythms through coordinated actions on multiple 5‐HT receptor subtypes.^52^ 2‐Glucosyl‐α‐glucoside is hydrolyzed to glucose and subsequently enters cellular energy metabolism pathways, thereby ensuring a consistent glucose supply to smooth muscle cells. Meanwhile, glyceraldehyde—a key intermediate in the glycolytic pathway—directly contributes to energy generation by facilitating ATP synthesis, thus supporting sustained smooth muscle contraction. Succinate directly enhances contraction efficiency by activating the calcium signaling pathway, which serves as a key regulator for smooth, skeletal, and cardiac muscle contraction.^53^ Therefore, *C. aurantium* honey presents a more favorable option for managing multifactorial GMDS.

In terms of anti‐inflammatory effects and intestinal barrier protection, the single component hesperidin demonstrates limited efficacy in intestinal barrier repair. Its primary activities are manifested through anti‐inflammatory and antioxidant actions, whereas its effectiveness in structural reconstruction and functional restoration of the barrier remains comparatively weak.^54^ *C. aurantium* honey forms an anti‐inflammatory network through its multiple components, whereas panose reduces endotoxin release by modulating gut microbiota composition.^55^ Oleamide suppresses mast cell degranulation, thereby reducing the release of pro‐inflammatory mediators.^56^ Diocyl succinate attenuates pro‐inflammatory cytokine levels through suppression of the NF‐κB signaling pathway.^57^ Collectively, these components act synergistically to enhance intestinal epithelial tight junction integrity, thereby effectively disrupting the “inflammation‐motility inhibition” pathological cycle.

These findings demonstrate that *C. aurantium* honey provides novel directions and candidate targets for mechanistic investigation and drug development related to GMDS and intestinal inflammatory conditions.

There are some limitations in this study. First, the analytical methodology exhibits constraints: the UHPLC–MS/MS analysis demonstrated suboptimal separation of hydrophobic metabolites, and the absence of overlaid blank controls limits the evaluation of baseline noise, collectively affecting the integrity of metabolite identification that underpins the network pharmacology predictions for *C. aurantium* honey. Second, although the *Drosophila* model offers valuable mechanistic insights, the lack of mammalian validation precludes definitive conclusions regarding its efficacy and mechanism in more physiologically complex systems, thereby restricting the direct translational relevance of the findings. Third, a critical mechanistic dimension remains unexplored—the role of the gut microbiota. As *C. aurantium* honey contains potential prebiotic components, the omission of microbiota profiling precludes determining whether the observed intestinal regulatory and anti‐inflammatory effects are mediated directly, indirectly through microbial modulation, or via a synergistic component‐microbiota‐host axis, resulting in an incomplete mechanistic framework. Finally, a technical limitation exists in the in vivo gut motility assay, wherein individual fly intake of the fluorescent tracer was not quantified, potentially compromising the reproducibility and quantitative precision of the measurements.

## 5 CONCLUSIONS

Our work expands the understanding of *C. aurantium* honey beyond a nutritional supplement to a systems‐level regulator of gut homeostasis. We have uncovered how it coordinately fine‐tunes the TORC1 signaling pathway to restore motility while concurrently mitigating oxidative stress and reinforcing the intestinal barrier. Our findings highlight a scientifically grounded framework for using this natural product to manage complex gastrointestinal disorders characterized by intertwined dysmotility and inflammation.

## AUTHOR CONTRIBUTIONS

Wenqi Wan and Ruiguang Huang contributed equally. Zhiyong Liu and Wenqi Wan designed the experiments. Wenqi Wan and Ruiguang Huang performed the in vivo experiments. Wenkai Zhang performed the UHPLC‐Q Exactive HF‐MS. Shuqiong Cao and Luxia Pan helped with the data analysis. Zhiyong Liu and Wujun Jiang provided funding. All authors reviewed the results and approved the final version of the manuscript.

## FUNDING INFORMATION

This work was partially supported by Jiangxi Human Resources and Social Security Department Expert Service Team Project (no.: 2025‐94‐6).

## CONFLICT OF INTEREST STATEMENT

The authors declare no conflicts of interest.

## ETHICS STATEMENT

Experiments were conducted in accordance with institutional guidelines and were approved by the Experimental Animal Science and Technology Center of JXUTCM. Ethics approval number is JZLLSC20250595.

## Data Availability

The datasets used and/or analyzed during the current study are available from the corresponding author on reasonable request.
